# Identification and control for the effects of bioinformatic globin depletion on human RNA-seq differential expression analysis

**DOI:** 10.1038/s41598-023-28218-7

**Published:** 2023-02-01

**Authors:** Dylan Sheerin, Francisco Lakay, Hanif Esmail, Craig Kinnear, Bianca Sansom, Brigitte Glanzmann, Robert J. Wilkinson, Matthew E. Ritchie, Anna K. Coussens

**Affiliations:** 1 Infectious Diseases and Immune Defence Division, The Walter and Eliza Hall Institute of Medical Research, 1G Royal Parade, Parkville, VIC 3052 Australia; 2 Department of Medical Biology, The University of Melbourne, Parkville, VIC Australia; 3 Wellcome Centre for Infectious Diseases Research in Africa and Institute of Infectious Disease and Molecular Medicine, University of Cape Town, Observatory, Cape Town, Western Cape South Africa; 4 MRC Clinical Trials Unit at University College London, Institute of Clinical Trials and Methodology, London, WC1V 6LJ UK; 5 Institute for Global Health, University College London, London, WC1E 6JB UK; 6 South African Medical Research Council Genomics Centre, Francie Van Zijl Drive, Parow Valley, Cape Town, Western Cape South Africa; 7 Francis Crick Institute, London, NW1 1AT UK; 8 Epigenetics and Development Division, The Walter and Eliza Hall Institute of Medical Research, 1G Royal Parade, Parkville, VIC 3052 Australia; 9 Imperial College London, SW7 2AZ, London, UK; 10 Present Address: Vuka Research Clinic, University of Cape Town, Department of Medicine, 8 Mzala Street, Khayelitsha, Cape Town, Western Cape South Africa

**Keywords:** Data processing, Quality control, Statistical methods, Tuberculosis

## Abstract

When profiling blood samples by RNA-sequencing (RNA-seq), RNA from haemoglobin (Hgb) can account for up to 70% of the transcriptome. Due to considerations of sequencing depth and power to detect biological variation, Hgb RNA is typically depleted prior to sequencing by hybridisation-based methods; an alternative approach is to deplete reads arising from Hgb RNA bioinformatically. In the present study, we compared the impact of these two approaches on the outcome of differential gene expression analysis performed using RNA-seq data from 58 human tuberculosis (TB) patient or contact whole blood samples–29 globin kit-depleted and 29 matched non-depleted—a subset of which were taken at TB diagnosis and at six months post-TB treatment from the same patient. Bioinformatic depletion of Hgb genes from the non-depleted samples (bioinformatic-depleted) substantially reduced library sizes (median = 57.24%) and fewer long non-coding, micro, small nuclear and small nucleolar RNAs were captured in these libraries. Profiling published TB gene signatures across all samples revealed inferior correlation between kit-depleted and bioinformatic-depleted pairs when the proportion of reads mapping to Hgb genes was higher in the non-depleted sample, particularly at the TB diagnosis time point. A set of putative “globin-fingerprint” genes were identified by directly comparing kit-depleted and bioinformatic-depleted samples at each timepoint. Two TB treatment response signatures were also shown to have decreased differential performance when comparing samples at TB diagnosis to six months post-TB treatment when profiled on the bioinformatic-depleted samples compared with their kit-depleted counterparts. These results demonstrate that failure to deplete Hgb RNA prior to sequencing has a negative impact on the sensitivity to detect disease-relevant gene expression changes even when bioinformatic removal is performed.

## Introduction

Haemoglobin (Hgb) RNAs originate from reticulocytes which typically exceed the proportion of leukocytes in circulation and can therefore contribute between 50 and 70% of the mRNA population sampled from whole blood^1^. Depending on the depth of RNA-sequencing (RNA-seq) achieved per sample, highly abundant reads originating from Hgb RNA can reduce sensitivity to detect lower-level transcripts in the blood transcriptome^2^. The inclusion of Hgb RNAs in library preparations has an impact on sequencing costs as greater sequencing depths are required to characterise these lower abundance transcripts. As a result, most researchers working with blood-derived RNA opt for methods to remove Hgb RNA upstream of library preparation by either RNAse-H enzymatic depletion or, more conventionally and efficiently, probe hybridisation^3^. An alternative method that has been suggested is the bioinformatic depletion of reads arising from globin transcripts, which has been proposed to adequately deal with this artefact of RNA processing^4^.

Globin-depletion prior to sequencing has been shown to improve the quality of RNA-seq data and enhance detection of biological variation^5,6^. Based on these findings it is tempting to speculate that the removal of such a large percentage of transcripts that are typically of little-to-no interest would have a substantial impact on the abundance and quantity of biologically relevant genes sequenced and reduction in technical variation between samples in a given experiment. However, the only published study that has compared differentially expressed genes (DEG) between libraries prepared with and without Hgb RNA depletion was a small patient:control study, consisting of three sarcoid patients (average age 59) and three healthy controls (average age 28); Harrington et al. found no statistically significant increase in the number of DEG identified when using kit-based Hgb RNA depletion compared to bioinformatic Hgb RNA depletion^4^.

Given the small sample size and distinct biological phenotypes of the two comparison groups used by Harrington et al.^4^, we aimed to overcome these limitations by using a larger sample size and assessing the impact of globin-depletion methods on the detection of paired longitudinal transcriptional changes in a clinical cohort. We thus compared 29 matched paired-end mRNA-seq libraries prepared with and without Hgb RNA depletion, and compared performance of paired differential expression analysis of blood transcriptome changes in persons living with HIV at the time of tuberculosis (TB) diagnosis and following six months of standard TB treatment. As blood Hgb levels are known to exhibit sex bias, with females having ~ 12% lower levels than males on average^7^, we factored this into our sample selection for the analysis. Profiling published TB gene signatures on both sets of samples we investigate the relationship between the proportion of reads mapping to Hgb genes and the correlation in enrichment scores between pairs of kit-depleted and non-depleted samples, the relationship to participant sex, as well as the performance of scores between the two types of samples. We also compared the profile of DEG detected between TB diagnosis (D0) and end of treatment (M6), for samples globin-depleted pre-library preparation to those bioinformatically globin-depleted post-sequencing, identifying a set of “globin-fingerprint” genes for each time point which can be used to estimate the impact of the library preparation method on the data. Overall, our findings demonstrate a negative impact of including Hgb RNA on the sensitivity to detect disease-relevant biological variation, despite bioinformatic removal of these transcripts.

## Results

### Highly abundant globin transcripts alter the composition of human blood RNA-seq libraries

**Figure 1:**
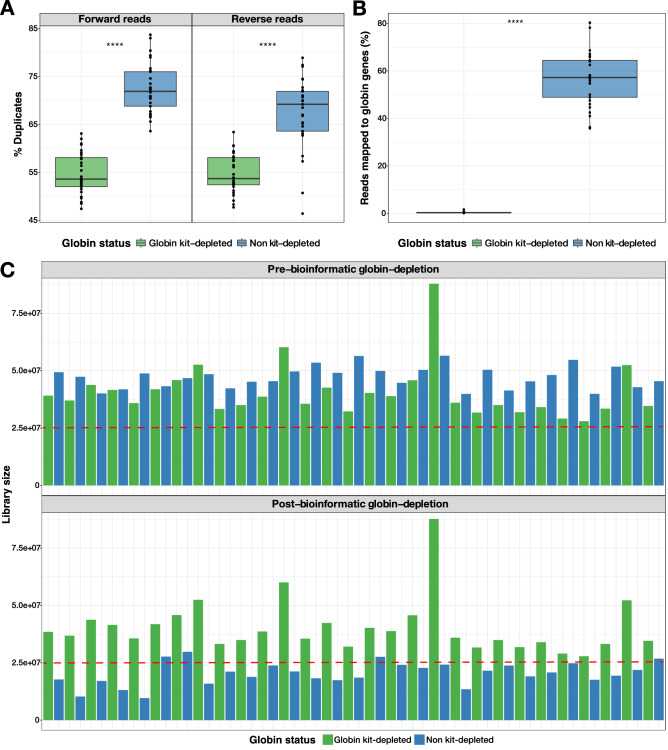
Sequencing RNA without globin-depletion profoundly alters the composition of resultant libraries. (A) Percentage of duplicated reads in forward and reverse reads from globin kit-depleted (green, *n* = 29) and non-depleted (blue, *n* = 29) samples. (**B**) Percentage of reads mapping to globin genes. Box plots indicate the median, 1st quartile and 3rd quartile while the whiskers indicate the lower and upper adjacent values. A paired *t* test was performed to compare each group of samples with and without prior globin depletion and the Bonferroni-corrected *p* values are displayed; ****p < 0.00005. (**C**) Library sizes for the 29 duplicated samples before (upper panel) and after (lower panel) bioinformatic globin-depletion. The horizontal dashed red line indicates a threshold library size of 2.5 × 10^7^ gene counts.

Samples selected for RNA-seq were from eight female and eight male study participants, at multiple time points. A total of 58 samples–29 non-depleted and 29 matched globin kit-depleted samples from the same whole blood mRNA specimens were used (Supplementary Table 1). Total read counts for the globin kit-depleted libraries across 29 samples after gene quantification was 1,174,727,310 compared with 1,368,387,638 for the 29 non-depleted libraries. The total read counts for D0 and M6 samples in the globin-depleted libraries were 443,110,507 and 446,487,562, respectively; whilst D0 and M6 read counts totalled 552,356,337 and 498,669,208, respectively, for the non-depleted samples. Prior to bioinformatic globin-depletion, non-depleted samples were associated with a significantly higher percentage of duplicate reads compared with the globin kit-depleted samples (*p* < 2.2 × 10^−16^) (Fig. 1A). A greater percentage of duplicates for the non-depleted samples was observed for the forward reads (median = 71.9%, *p* < 2.2 × 10^−16^) compared with reverse reads (median = 69.2%, *p* = 5.7 × 10^−9^, Fig. 1A). Despite the similarity in total read counts for the two library preparation methods, the proportion of globin reads in non-depleted samples ranged from 35.84 to 80.33% (median = 57.24%), compared to 0.15–1.62% for kit-depleted samples (median = 0.32%, *p* = 6.3 × 10^−10^) (Fig. 1B). To perform differential gene expression (DGE) analyses, reads mapping to globin genes—*HBA1*, *HBA2*, *HBB*, *HBBP1*, *HBD*, *HBE1*, *HBG1*, *HBG2*, *HBM*, *HBQ1*, *HBZ* and *HBZP1* – were bioinformatically removed from all samples prior to normalisation, resulting in a large reduction in library size for the non-depleted samples (Fig. 1C) – decreasing from 1,368,387,638 total reads to 588,272,669 usable reads, representing a 57% drop following bioinformatic globin-depletion. By comparison, a reduction of just 0.37% usable reads was seen for the globin kit-depleted libraries before and after bioinformatic globin-depletion. Before bioinformatic depletion all samples had a library size above the threshold read count of 2.5 × 10^7^, whilst only four of the 29 non-depleted samples remained above this threshold after bioinformatic depletion (Fig. 1C). As the bioinformatic globin depletion had minimal effect on the library size of the kit-depleted samples, for simplicity we will hereafter refer to these samples as kit-depleted and the other samples as bioinformatic-depleted.

**Figure 2:**
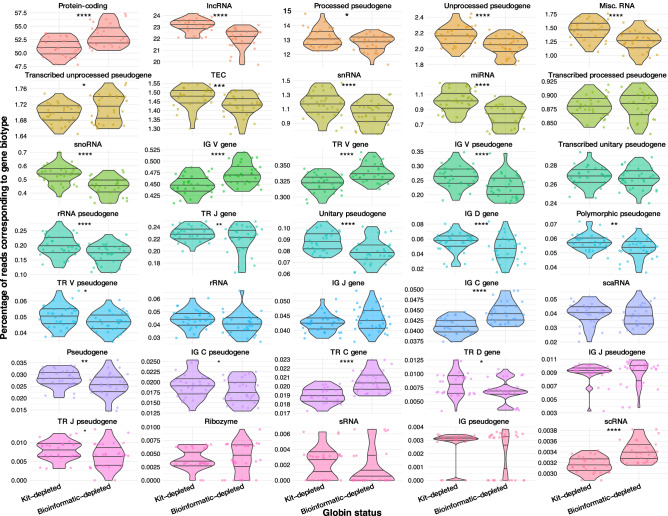
Distributions of reads mapping to distinct gene biotype regions of the genome between kit- and bioinformatic-depleted samples. Violin plots depicting the distribution of the proportion of reads from each library method (*n* = 29 samples per method) mapping to portions of the genome associated with specific gene biotypes. Percentages were calculated after the bioinformatic removal of haemoglobin protein-coding genes from all samples. Each dot represents and individual sample from each library method. Horizontal lines indicate the median, 1st quartile and 3rd quartile while the ends of the “violin” indicate the lower and upper adjacent values. A paired *t* test was performed to compare each group of samples with and without prior globin depletion and the Bonferroni-corrected *p* values are displayed; * < 0.05, ** < 0.005, **** < 0.00005.

To investigate whether transcripts of lower abundance or smaller transcript size were preferentially underrepresented in the bioinformatic-depleted libraries, gene biotype frequencies were compared between kit-depleted and bioinformatic-depleted samples using the normalised dataset (Fig. 2). We found a significantly higher proportion of the reads mapping to non-haemoglobin protein-coding genes in the bioinformatic-depleted samples (median, 53%; interquartile range, 2.8), compared to those kit-depleted (median, 51%; interquartile range, 2.3), and a relative underrepresentation of other gene biotypes. This occurred as a result of the dramatic reduction in library sizes of bioinformatic-depleted samples and indicates a loss of gene biotype diversity relative to the kit-depleted samples.

For non-protein-coding gene biotypes that were represented in greater than 1% of reads, kit-depleted samples had a significantly higher % of reads detected for all these biotypes, with the exception of those mapping to transcribed unprocessed pseudogenes. Overall, bioinformatic-depleted samples had lower proportions of reads mapped to regions of the genome from which smaller RNA species, such as long non-coding (lncRNA), micro (miRNA), small nuclear (snRNA) and small nucleolar RNAs (snoRNA), are derived; indicating a differential impact of sequencing Hgb RNA on the ability to detect genes involved in post-transcriptional regulation. It should be noted that mRNA was purified from total RNA prior to library preparation, so the proportions of these RNA species may reflect inefficient polyA selection. However, as the samples were all prepared by the same method, these results still indicate significant differences in the proportions of small RNAs that are sequenced when globin is depleted before library preparation. It is likely that the impact on small RNA detection may be even more apparent when total RNA is sequenced. A more varied trend was observed for reads mapping to portions of the genome encoding immunoglobulin (IG) and T cell receptor (TR) subunits, with IG D genes, IG pseuodegenes, TR J genes, TR V pseudogenes, IG C pseudogenes, TR D genes and TR J pseudogenes accounting for a significantly higher proportion of genes in kit-depleted samples whilst IG V genes, TR V genes, IG C genes and TR C genes exhibited the opposite pattern (Fig. 2).

### Bioinformatic removal of globin reads does not uniformly restore differential gene expression detection sensitivity

**Figure 3:**
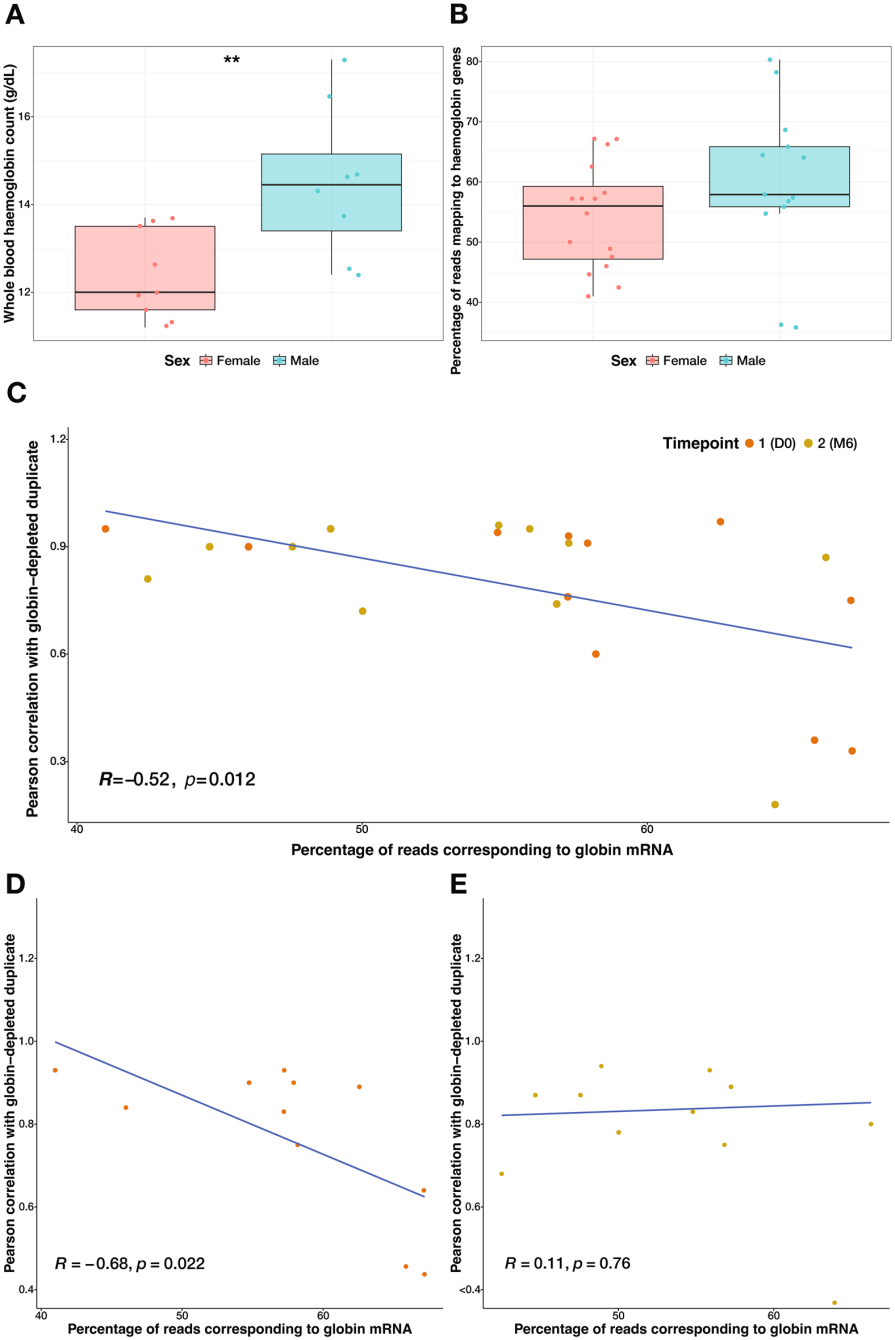
Non-depleted samples do not uniformly correlate with kit-depleted duplicates. (**A**) Whole blood haemoglobin counts for a subset of samples [*n* = 9 female (red), *n* = 8 male (blue)] measured in grams per decilitre (g/dL). Box plots indicate the median, 1st quartile and 3rd quartile while the whiskers indicate the lower and upper adjacent values. (**B**) Proportions of total reads mapping to haemoglobin genes in all 29 non-depleted samples prior to bioinformatic globin depletion, stratified by female and male. * < 0.05, Wilcoxon Rank Sum test. (**C**) Pearson correlation between the proportion of reads mapping to haemoglobin genes prior to bioinformatic globin depletion in the non-depleted samples and the Pearson correlation values derived from a correlation analysis of gene set variation analysis (GSVA) scores generated from 70 tuberculosis signatures between matched pairs of globin-depleted (*n* = 22) and non-depleted samples (*n* = 22). Each data point represents a matched pair at either TB diagnosis (D0, orange) or end of TB treatment (M6, mustard). The blue line represents the fitted linear regression model. (**D**–**E**) Pearson correlation between the proportion of reads mapping to haemoglobin genes in non-depleted samples prior to bioinformatic globin depletion and the Pearson correlation values derived from a correlation analysis of GSVA scores generated from 70 tuberculosis signatures between matched pairs of kit-depleted and bioinformatic-depleted samples. (**D**) Samples from the point-of-diagnosis of active tuberculosis (D0, *n* = 11). (**E**) Samples from 6 months post-tuberculosis treatment (M6, *n* = 11). Each data point represents a matched kit-depleted and bioinformatic-depleted pair. The blue line represents the fitted linear regression.

To determine whether the donor’s sex influenced their blood Hgb levels, whole blood Hgb counts were compared between males and females for whom these data were available at time of RNA blood sampling (*n* = 17 samples, nine female, eight male). Males had significantly higher Hgb counts in their blood than females in this subset of samples (Fig. 3A, p = 0.0093, Wilcoxon Rank Sum test). Plotting the proportion of reads mapping to Hgb genes between male and female samples indicated higher proportions in male samples compared with female (Fig. 3B, Wilcoxon Rank Sum test), but this difference was not found to be significant. Overall, no significant correlation was observed between the whole blood Hgb count and the corresponding proportion of reads mapping to Hgb genes (*R* = 0.18, *p* = 0.4857).

Having established the contribution of sex-specific differences in Hgb counts and reads, the next step was to assess the consistency of biologically relevant gene expression between kit-depleted and bioinformatic-depleted paired samples, a total of 70 published TB signatures were profiled on the normalised post-bioinformatic globin-depletion dataset of 58 samples using the gene set variation analysis (GSVA) function of the TBSignatureProfiler package^8^ (Supplementary Figure. 1). These signatures were derived from similar experiments comparing TB patient samples pre- versus post-treatment or TB patient samples with latent TB or healthy control samples and therefore represent sets of genes that we would expect to be differentially expressed, resulting in consistent GSVA scores between pairs of samples, in our dataset. GSVA scores across all 70 signatures were analysed by Pearson correlation between the 29 paired kit-depleted and bioinformatic-depleted paired samples. Pairwise correlation values ranged from 0.31 to 0.96 (median 0.88) across all samples; correlation values were marginally higher for D0 [0.62–0.96 (median 0.89)] compared to M6 [0.31–0.96 (median 0.88)] samples. Of the 70 signatures tested, 12 had significantly different GSVA scores between paired kit-depleted and bioinformatic-depleted duplicates, indicating the performance of some TB signatures varies depending on sample processing method used to acquire RNA-seq data. In general, poor score correlation was seen for signatures derived from large (> 80) and small (< 15) numbers of genes (Supplementary Figure 1). To highlight the genes in these signatures that exhibited the greatest discordance between kit-depleted and bioinformatic-depleted pairs, heatmaps were generated for the 12 signatures that resulted in significant differences between GSVA scores (Supplementary Figure 2).

To determine to what extent poor correlation was due to globin transcript abundance, a Pearson correlation was performed on samples with matched timepoint data and a consistent biological background (HIV+ only) (Supplementary Table 1); Pearson correlation values from the GSVA were correlated with the proportion of reads mapping to globin genes in the non-depleted sample prior to bioinformatic depletion (Fig. 3C). A moderate, significant, inverse correlation was observed (*R* = -0.52, *p* = 0.013), demonstrating that a high proportion of reads mapping to globin genes in a given sample can lead to poor conservation of the core biological expression profile for that sample. Pearson correlation values were also correlated with library size post-bioinformatic depletion, revealing a moderate but non-significant correlation (*R* = 0.28, *p* = 0.14) that indicates this trend cannot be explained by sequencing depth (Supplementary Figure 3). Separating samples based on clinical status, D0 or M6 (Fig. 3D–E), revealed a greater inverse correlation at the TB diagnosis timepoint (D0, *R* =  − 0.68, *p* = 0.022) and no significant correlation in samples taken 6 months post-therapy timepoint (M6, *R* =  − 0.11, *p* = 0.76). Previous studies have shown that the blood transcriptional perturbation present at the time of TB diagnosis normalises to healthy levels after 6 months of treatment^9^. This suggests a greater impact of Hgb contamination on the consistency of TB signature profiling at timepoints where greater transcriptional perturbation and therefore higher TB signature GSVA scores are observed.

### “Globin fingerprints” can be detected in bioinformatic-depleted samples and may reduce sensitivity to detect biological variation

**Figure 4:**
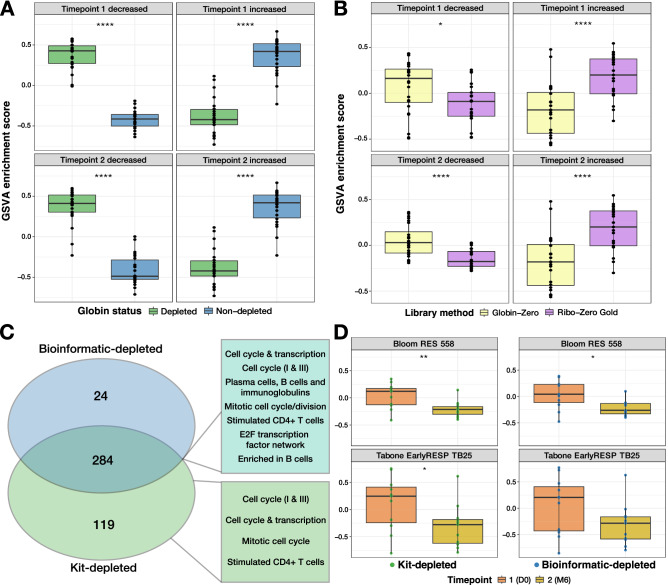
Enrichment of “globin fingerprints” may have a negative impact on sensitivity to detect differential expression and biomarker performance. (**A**) Gene set variation analysis (GSVA) of globin fingerprint signatures derived from significantly increased and decreased genes transcript abundances identified by contrasting kit-depleted and bioinformatic-depleted samples at two biologically distinct timepoints [Timepoint 1, tuberculosis (TB) diagnosis (D0); Timepoint 2, end of TB treatment (M6)]. Significantly differentially expressed genes were defined as those with false discovery rate (FDR)-adjusted *p* values below 0.05 and a log_2_ fold-change (LFC) greater than 1.5. Signatures were filtered to include only the top 200 genes according to their FDR-adjusted *p* values. Box plots indicate the median, 1st quartile and 3rd quartile while the whiskers indicate the lower and upper adjacent values. A *t* test was performed to compare each group of samples with and without prior globin depletion and the Bonferroni-corrected *p* values are displayed; * < 0.05, ** < 0.005, **** < 0.00005. (**B**) Validation of “globin signatures” from (**A**) on a similar dataset from a patient:control study from Harrington et al.^4^. **C.** Venn diagram depicting the overlap in significantly differentially expressed genes identified separately for kit-depleted and bioinformatic-depleted samples by D0 samples with matched samples from M6. Module terms listed to the right of intersections were generated by performing a blood transcriptional module analysis on the corresponding genes. (**D**) GSVA analysis performed on the TB dataset using published and validated TB treatment response signatures from Bloom et al.^9^ and Tabone et al.^10^. The left panels correspond to kit-depleted samples and the right to bioinformatic-depleted samples. Bonferroni-corrected *p* values are displayed; * < 0.05, ** < 0.005, **** < 0.00005.

To determine the specific effects of sequencing globin mRNA on resultant DGE analyses, the kit-depleted and bioinformatic-depleted samples were directly contrasted using edgeR quasi-likelihood testing. The paired samples were first split into D0 and M6 timepoints (*n* = 11 kit-depleted and *n* = 11 bioinformatic-depleted, per timepoint) so that comparisons were performed between samples of a similar biological state. This ensured that differential gene transcript abundance captured by these comparisons would be related to technical variation from the library preparation method rather than biological variation driven by the underlying disease state. Many of the genes with decreased transcript abundance in kit-depleted samples relative to bioinformatic-depleted samples (and therefore increased abundance in bioinformatic-depleted samples) were those encoding ribosomal proteins (Supplementary Table 2). This was observed despite no significant differences being detected in the proportion of ribosomal transcripts between kit-depleted and bioinformatic-depleted libraries (Fig. 2). The top 200 significantly increased and decreased DEG derived at each timepoint (Supplementary Table 2) were then profiled by GSVA on all 29 paired samples, irrespective of timepoint (Fig. 4A). DEG identified to differ between kit-depleted and bioinformatic-depleted samples at either timepoint performed equally well at differentiating kit-depleted and bioinformatic-depleted samples, when all 29 paired samples were compared. Identification of these “globin fingerprint” DEG during DGE analysis may serve as a useful indication of whether samples have been significantly compromised by the inclusion of Hgb RNA prior to library preparation.

To validate the performance of the “globin fingerprint” to detect artefacts of sequencing libraries without prior removal of Hgb RNA, the same GSVA was performed on the Harrington et al. dataset^4^. Samples from both sarcoidosis patients and healthy controls were grouped together according to their library method (Globin-Zero or Ribo-Zero Gold (which does not deplete globin mRNA) to assess the impact of technical variation, irrespective of disease status. The GSVA exhibited the same pattern of variation between globin-depleted and non-depleted groups as the TB dataset (Fig. 4B), indicating strong performance of these “globin fingerprint” signatures on biologically distinct human samples.

### Lack of globin-depletion pre-processing reduces sensitivity to detect biologically significant gene expression differences

To determine what is lost in terms of DEG for the biological comparison of interest between timepoints, another pair of contrasts were made between the D0 and M6 timepoints for the kit-depleted samples and bioinformatic-depleted samples, separately. Of the 406 significantly DEG identified across all samples for this contrast, 284 (~ 70%) were shared between the library methods, 98 (~ 24%) were exclusively identified in the kit-depleted samples and 24 (5.9%) were unique to the bioinformatic-depleted samples (Fig. 4C). A modular analysis of the shared DEG revealed enrichment of modules associated with B cells (LI.M156.1 *p* < 2 × 10^−16^, LI.M156.0 *p* = 1.1 × 10^−14^, LI.M47.2 *p* = 0.00059) as well as transcription regulation and cell cycle. DEG that were only detected in the kit-depleted samples were also enriched for cell cycle modules (LI.M4.1 *p* = 1.4 × 10^−6^, LI.M4.0 *p* = 1.5 × 10^−6^, LI.M103 *p* = 8.1 × 10^−6^) and a stimulated CD4^+^ T cell module (LI.M46 *p* = 3.7 × 10^−5^). Conversely, DEG uniquely identified in the bioinformatic-depleted samples were not significantly enriched for any module. This indicates that some key biological pathways associated with TB treatment could not be detected in samples that had not undergone globin kit-depletion and therefore key information was missing due to the loss of sensitivity. Neither inclusion of the blood Hgb levels nor percentage of reads mapping to Hgb RNA as a covariate in the models used for DEG testing had any effect on restoring the DGE call for bioinformatic-depleted samples.

Finally, in order to assess whether signatures of TB treatment derived from other datasets performed equally well on kit-depleted and bioinformatic-depleted samples, a pair of TB treatment signatures identified by Bloom et al.^9^ and Tabone et al.^10^ were profiled by GSVA on the TB dataset. The Bloom et al. signature was less significantly different between D0 and M6 in the bioinformatic-depleted samples compared with the kit-depleted samples, while the Tabone et al. signature was not significantly different between D0 and M6 for the bioinformatic-depleted samples whilst it was significantly different in the kit-samples (Fig. 4D). This demonstrates that failure to globin kit-deplete RNA samples prior to RNA-seq library preparation may lead to sub-optimal performance of validated biomarker signatures on datasets in which they may otherwise perform well.

### Discussion

We present the results of an assessment of the effects of including Hgb RNA in the sequencing of RNA and compare two approaches of dealing with Hgb in such experiments —  bioinformatic removal of Hgb reads after the sequencing run or hybridisation-based removal of Hgb RNA prior to library preparation. Bioinformatic depletion of reads mapping to Hgb genes had a substantial impact on the overall library size and impacted on the proportion of small RNA species recovered. This had further impact on the subsequent DGE call when comparing samples from TB patients pre- and post-drug treatment.

Our analysis expands upon prior assessments of the effects of globin depletion in a number of key aspects. Initial assessments of the benefits of globin removal prior to sequencing demonstrated the benefit of hybridisation-based globin depletion methods to improve the detection of low-abundance transcripts compared with non-depleted samples^5,6,11^; however, these analyses were restricted to polyA-selected libraries and therefore exclusively mRNA. We elected to sequence total RNA to determine whether there was any further benefit to non-coding RNA and whether the benefits observed for globin depletion of polyA libraries would still hold for libraries generated using a method that depletes ribosomal RNA. Most importantly, our analysis offers several improvements over a recent study that also took this factor into account^4^. Our sample size was greater, allowing us to assess differences between female and male participants as well comparing disease-relevant changes in matched participants over two longitudinally sampled timepoints – active TB diagnosis and six months post-TB treatment – facilitating a robust assessment of the impact of Hgb gene reads on DGE call. We also went a step further in this assessment and profiled the effects of a de novo derived “globin fingerprint” on the transcriptional response that should be observed in response to TB drug treatment.

The existence of sex differences in Hgb levels between females and males and the implications this has for the diagnosis of blood-based disorders has long been understood^12,13^. Hgb levels are also negatively associated with erythropoietin levels in the blood, indicating that the sex difference is constitutive^14^. This difference may play a physiological role in determining differential outcomes of disease such as sepsis and stroke^15,16^. In terms of infectious diseases, a protective role for Hgb C has been determined for *Plasmodium falciparum* infection in malaria endemic regions^17–19^. However, transcriptional differences in Hgb subunit-encoding genes are not typically studied in the context of disease and, where Hgb levels are a parameter of interest it is often blood counts that are assessed. Given the proportion of sequencing space that is taken up by Hgb gene reads when they are not removed prior to library preparation for RNA-seq, sex differences and potential differences in Hgb levels between patients and healthy control participants may be an important consideration for experimental design. In our analysis, nine female and eight male participants showed significant differences in blood Hgb counts and, while the difference in reads corresponding to Hgb mRNA was not found to be significant the same trend of higher levels in males was observed. The impact of bioinformatic removal on library sizes may therefore have a differential impact on female and male samples, particularly if they vary significantly in Hgb levels during a given disease state, which could skew results of DGE analysis depending on the experimental design.

A study by Holik et al. assessing the impacts of two different RNA library preparation methods — one using polyA selection and another using ribodepletion – on various RNA-seq quality control metrics^20^. They observed differential recovery of small RNA species, including non-coding, small nuclear and small nucleolar RNAs, between the two methods with lower recovery of these species associated with the polyA kit. We found the inclusion of Hgb RNA to have the same impact on recovery of these small RNA species, with the additional effect of reducing miRNA recovery. This has important implications for experiments where the effects of non-coding RNA and post-transcriptional regulation are of interest. Increased recovery of Immunoglobiun and TCR genes was observed for bioinformatic-depleted samples. However, this may represent an artificial increase resulting from the inclusion of Hgb gene reads and may lead to the aberrant detection of such genes as significantly differentially expressed depending on the proportions of Hgb RNA between experimental groups. In terms of the impact on protein-coding genes, we observed a reduction of close to 60% in the total library size of bioinformatic-depleted samples compared with < 0.5% in the kit-depleted samples. This substantial loss of reads had consequences for the DGE call, with approximately 25% of total DEG not being detected for a given comparison of pre- and post-tuberculosis treatment samples. This is in stark contrast to the findings reported by Harrington et al.^4^ which conclude that of sequencing Hgb RNA and depleting reads bioinformatically has minimal impact on DGE call.

It has been suggested that inclusion of a quantifiable metric, such as RNA integrity number, as a linear model covariate for DGE testing can correct for most of the deleterious effects of sub-optimal libraries^21^. In our analysis, however, neither inclusion of the blood Hgb levels nor percentage of reads mapping to Hgb RNA had any effect on restoring the DGE call for bioinformatic-depleted samples. It is likely that increasing the sequencing depth could avert this impact, but this raises further considerations for cost/labour of Hgb RNA depletion prior to sequencing versus increased costs of sequencing. By first defining the putative Hgb-related effects on our dataset and profiling our derived “globin fingerprint” on the Harrington et al. dataset^4^, we identified significant enrichment of this gene set on both datasets. The genes identified may prove to be a useful resource for other researchers trying to gauge the success of bioinformatic globin depletion on their datasets with detection of these genes during DGE likely related to technical rather than biological variation.

Finally, further assessment of the impact of lower sensitivity to detect DEG in our dataset revealed a failure to detect several pathways involved in cell cycle and T cells as significantly enriched. Then by profiling the effects of known TB treatment response signatures^9,10^ on our data, with and without globin depletion prior to sequencing, we were able to assess the broader impact of including Hgb RNA in the sequencing run. The poorer performance of both signatures on the bioinformatic-depleted samples suggests that genes implicated in the biological response of interest are detected with reduced sensitivity when more of the sequencing space is taken up by Hgb RNA.

## Methods

### Sample collection and human ethics

Samples used for this study were derived from three TB cohort studies with ethical approval provided by the research ethics committee of the University of Cape Town (013/2011, 449/2014, 816/2016). All participants were residents of Khayelitsha, a peri-urban township of Cape Town, South Africa. Informed consent was sought from all participants prior to screening and phlebotomy. All research was performed in accordance with relevant guidelines/regulations.

3 mL of blood for RNA extraction was collected in Tempus tubes and stored in a freezer between − 20 °C and − 80 °C. EDTA blood was collected for full blood count which was performed in laboratories of the South African National Health Laboratory Services (NHLS) or the University of Cape Town.

### RNA extraction and quality control

RNA was extracted from thawed Tempus tubes using either the PerfectPure™ Blood RNA Kit (5 PRIME) or Norgen Preserved Blood RNA kits (for Tempus Tubes) (Norgen Biotek, ON, Canada) according to manufacturers’ instructions, including DNAse treatment. RNA integrity was assessed using the Bioanalyser Nano Assay (Agilent, Santa Clara, CA, US) with all samples having RNA integrity number (RIN) 7–10. RNA purity was assessed using a Nanodrop ND 1000 spectrophotometer (Thermo Fisher Scientific, MA, USA) and RNA yield was quantified using a Qubit RNA Board Range (BR) assay kit (Thermo Fisher Scientific).

### RNA-seq library preparation and sequencing

1000 ng of the isolated total RNA was globin reduced from 29 of the 58 samples using the GLOBINclear™—Human Kit (Invitrogen, MA, US). Dynabeads® mRNA DIRECT™ Kit (Invitrogen, MA, US) was used to purify mRNA from all 58 samples. Purified mRNA samples were quantified using the Qubit RNA BR assay kit (Thermo Fisher Scientific). 200 ng of RNA was converted to double-stranded cDNA (ds-cDNA). The ds-cDNA were subjected to heat denatured and circularised by the splint oligo sequence to generate the single stranded circle DNA followed by rolling circle replication to create DNA nanoball (DNB) using the MGIeasy RNA Library Prep (MGI Tech, Shenzhen, China). Samples were sequenced using the MGI DNBSEQ-G400RS (MGI Tech, Shenzhen, China), at two 100-base paired-end reads.

### Read alignment and quantification

Raw RNA-seq fastq files were subject to initial quality control analysis using FastQC (v0.11.9, https://www.bioinformatics.babraham.ac.uk/projects/fastqc/), generating a separate report for each forward and reverse read. These individual FastQC reports were consolidated into a single summary report using MultiQC (v1.12, https://multiqc.info/). A human genome index was built using the *Homo sapiens* GRCh38 DNA primary assembly file (http://ftp.ensembl.org/pub/release-106/fasta/homo_sapiens/dna/) using the Rsubread^22^ (v4.2) *buildindex* function. Forward and reverse reads were then aligned to the reference genome index using the Rsubread *align* function. Gene counts were quantified using the BAM files generated from this step, featureCounts^23^ (v2.0.3) and the *Homo sapiens* GRCh38.106 GTF file (http://ftp.ensembl.org/pub/release-106/gtf/homo_sapiens/).

### RNA-seq analysis and bioinformatic globin depletion

Count matrices from each batch of RNA-seq data were loaded into the RStudio^24^ (v1.4.1743–4, R 4.2.0) environment. The count matrix was used to create a DGEList object with edgeR^25^. Low expressed genes were filtered from the data using the edgeR *filterByExpr* function. The function’s default parameters retain genes with ≥ 10 counts in the user-defined minimum group size parameter, which in this instance was *n* = 3. Gene annotation was added to the DGEList with the “hsapiens_gene_ensembl” dataset obtained using the biomaRt^26^ package. Reads mapping to globin genes—*HBA1*, *HBA2*, *HBB*, *HBBP1*, *HBD*, *HBE1*, *HBG1*, *HBG2*, *HBM*, *HBQ1*, *HBZ* and *HBZP1*–were bioinformatically removed from all samples prior to normalisation. Sample normalisation factors were calculated in edgeR using the default trimmed mean of *m* values method. Three different approaches to DGE testing were taken—the first using a standard linear model for the experimental groups with no additional covariate, the second using blood Hgb levels as a linear model covariate, and the third using the proportion of reads mapping to Hgb RNA as a linear model covariate. These models were implemented using the edgeR quasi-likelihood pipeline described by Chen et al.^27^. A false discover rate (FDR) threshold of < 0.05 and an absolute log_2_ fold-change threshold of 1 were used to determine significant DE.

### Gene set variation analysis

Each signature of interest was used separately to generate an enrichment score by applying a gene set variation analysis (GSVA)^28^ with the TBSignatureProfiler package^8^. GSVA uses enrichment scoring which compares the rankings of the genes in a particular signature compared to all the other genes in that sample. Briefly, the composite gene list was mapped onto the gene expression statistics for each condition in the input dataset to generate a single enrichment summary statistic for each sample corresponding to increased or decreased expression of a TB resolution signature. TB resolution signature scores were compared between D0 and M6 using a two-sided *t* test, with Bonferroni correction to adjust for multiple testing.

## Supplementary Information

Supplementary Information 1.

Supplementary Information 2.

Supplementary Information 3.

## Data Availability

Unprocessed fastq files and the associated gene count matrices generated and/or analysed during the current study are available in the Gene Expression Omnibus (GEO) repository under accession number GSE215456. The R script used to analyse the data and generate manuscript figures is available from the GitHub repository, https://github.com/sheerind-wehi/RNA-seq-globin-analysis/blob/main/globin_DGE_analysis.R.
