# Supplementary material for: Identification and control for the effects of bioinformatic globin depletion on human RNA-seq differential expression analysis
Source: Sci Rep. 2023 Feb 1;13:1859. doi: 10.1038/s41598-023-28218-7 (PMC9892020; doi:10.1038/s41598-023-28218-7)
Supplement: Supplementary file 1 — Supplementary Information 1. [file 41598_2023_28218_MOESM1_ESM.pdf]

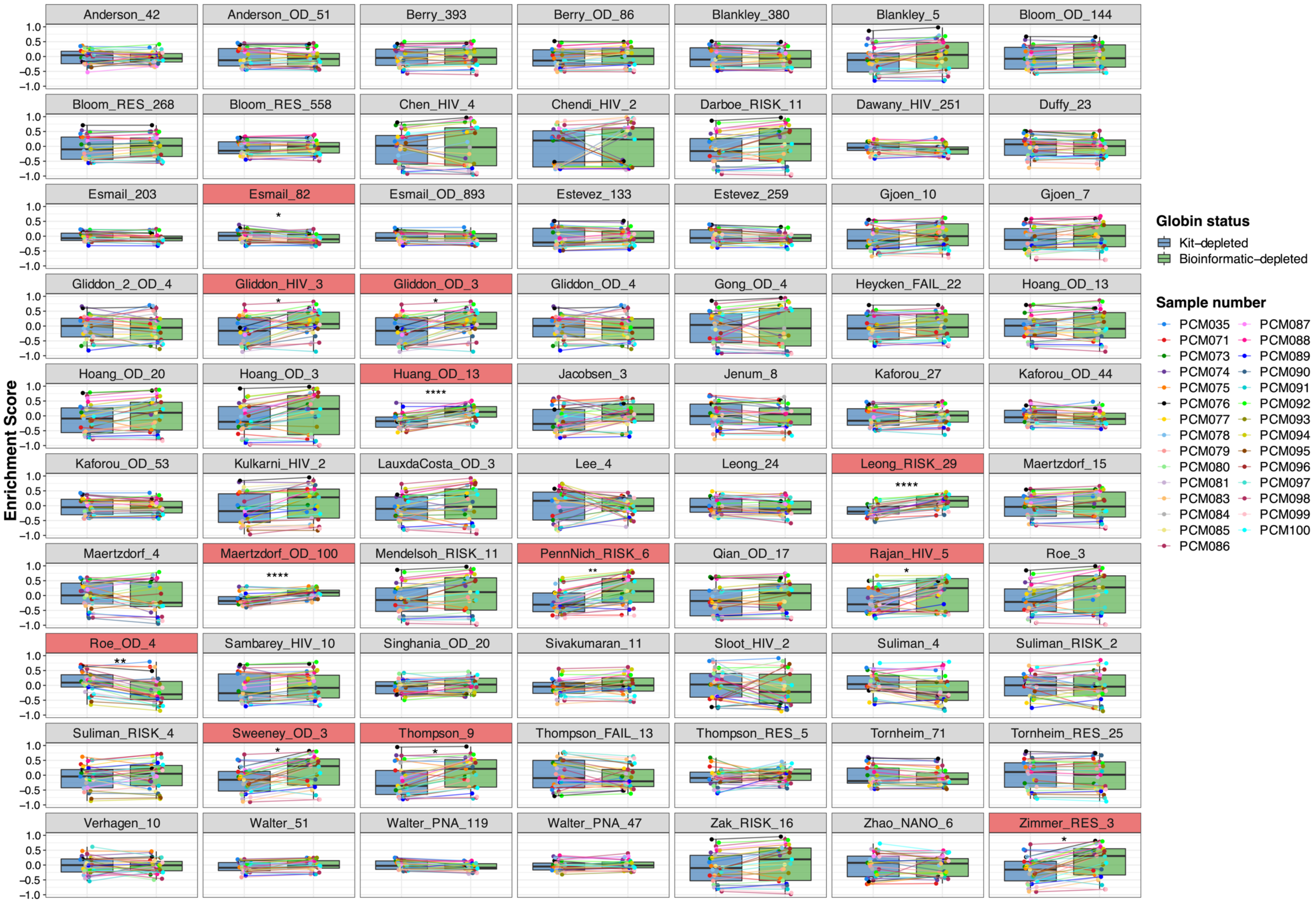

Enrichment Score
Anderson_42
Anderson_OD_51
Berry_393
Berry_OD_86
Blankley_380
Blankley_5
Bloom_OD_144
Bloom_RES_268
Bloom_RES_558
Chen_HIV_4
Chendi_HIV_2
Darboe_RISK_11
Dawany_HIV_251
Duffy_23
Esmail_203
Esmail_82
Esmail_OD_893
Estevez_133
Estevez_259
Gjoen_10
Gjoen_7
Gliddon_2_OD_4
Gliddon_HIV_3
Gliddon_OD_3
Gliddon_OD_4
Gong_OD_4
Heycken_FAIL_22
Hoang_OD_13
Hoang_OD_20
Hoang_OD_3
Huang_OD_13
Jacobsen_3
Jenum_8
Kaforou_27
Kaforou_OD_44
Kaforou_OD_53
Kulkarni_HIV_2
LauxdaCosta_OD_3
Lee_4
Leong_24
Leong_RISK_29
Maertzdorf_15
Maertzdorf_4
Maertzdorf_OD_100
Mendelsoh_RISK_11
PennNich_RISK_6
Qian_OD_17
Rajan_HIV_5
Roe_3
Roe_OD_4
Sambarey_HIV_10
Singhania_OD_20
Sivakumaran_11
Sloot_HIV_2
Suliman_4
Suliman_RISK_2
Suliman_RISK_4
Sweeney_OD_3
Thompson_9
Thompson_FAIL_13
Thompson_RES_5
Tornheim_71
Tornheim_RES_25
Verhagen_10
Walter_51
Walter_PNA_119
Walter_PNA_47
Zak_RISK_16
Zhao_NANO_6
Zimmer_RES_3
Globin status
Kit–depleted
Bioinformatic–depleted
Sample number
PCM035
PCM071
PCM073
PCM074
PCM075
PCM076
PCM077
PCM078
PCM079
PCM080
PCM081
PCM083
PCM084
PCM085
PCM086
PCM087
PCM088
PCM089
PCM090
PCM091
PCM092
PCM093
PCM094
PCM095
PCM096
PCM097
PCM098
PCM099
PCM100

Supplementary Figure 1. Gene set variation scores associated with each of 70 published tuberculosis (TB) signatures between kit-depleted and bioinformatic-depleted paired samples. Gene set variation analysis (GSVA) was performed using the TBSignatureProfiler package[8] in R to generate GSVA scores for 70 TB signatures from the published literature, curated by the package. Each signature is labelled by the first author of the signature and the number of genes in the signature. This analysis was performed on 29 kit-depleted (blue) and 29 matched bioinformatic-depleted (green) samples. Horizontal coloured lines link paired samples. Box plots indicate the median, 1st quartile and 3rd quartile while the whiskers indicate the lower and upper adjacent values. A paired t-test was performed to compare each group of samples with and without prior globin depletion and the Bonferroni-corrected p-values are displayed; *<0.05, **<0.005, ****<0.00005. Signatures with significant differences between the two groups are highlighted in red.

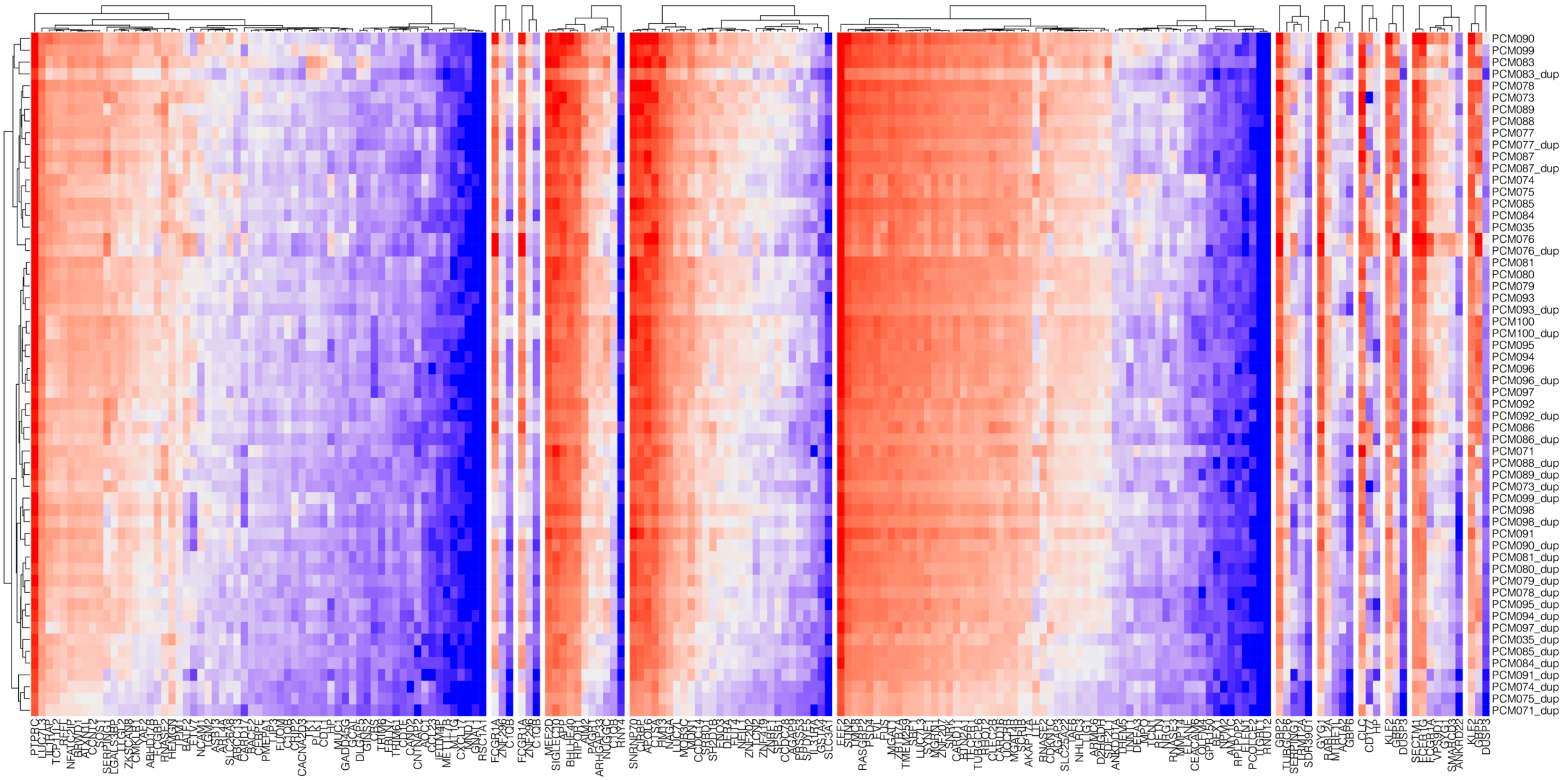

Supplementary Figure 2. Expression of tuberculosis (TB) signature genes across kit-depleted and bioinformatic-depleted samples. Constituent genes from each of the 12 TB signatures from the published literature found to be significantly differentially enriched between kit-depleted and bioinformatic-depleted samples were plotted across all samples as a heatmap. Samples are identified with PMC number and _dup indicates the matched sample that was globin kit-depleted. Z-scores were calculated for each gene and are represented by the colour of the tiles, with red being a higher positive z-score, blue a lower negative z-score and white a neutral z-score.

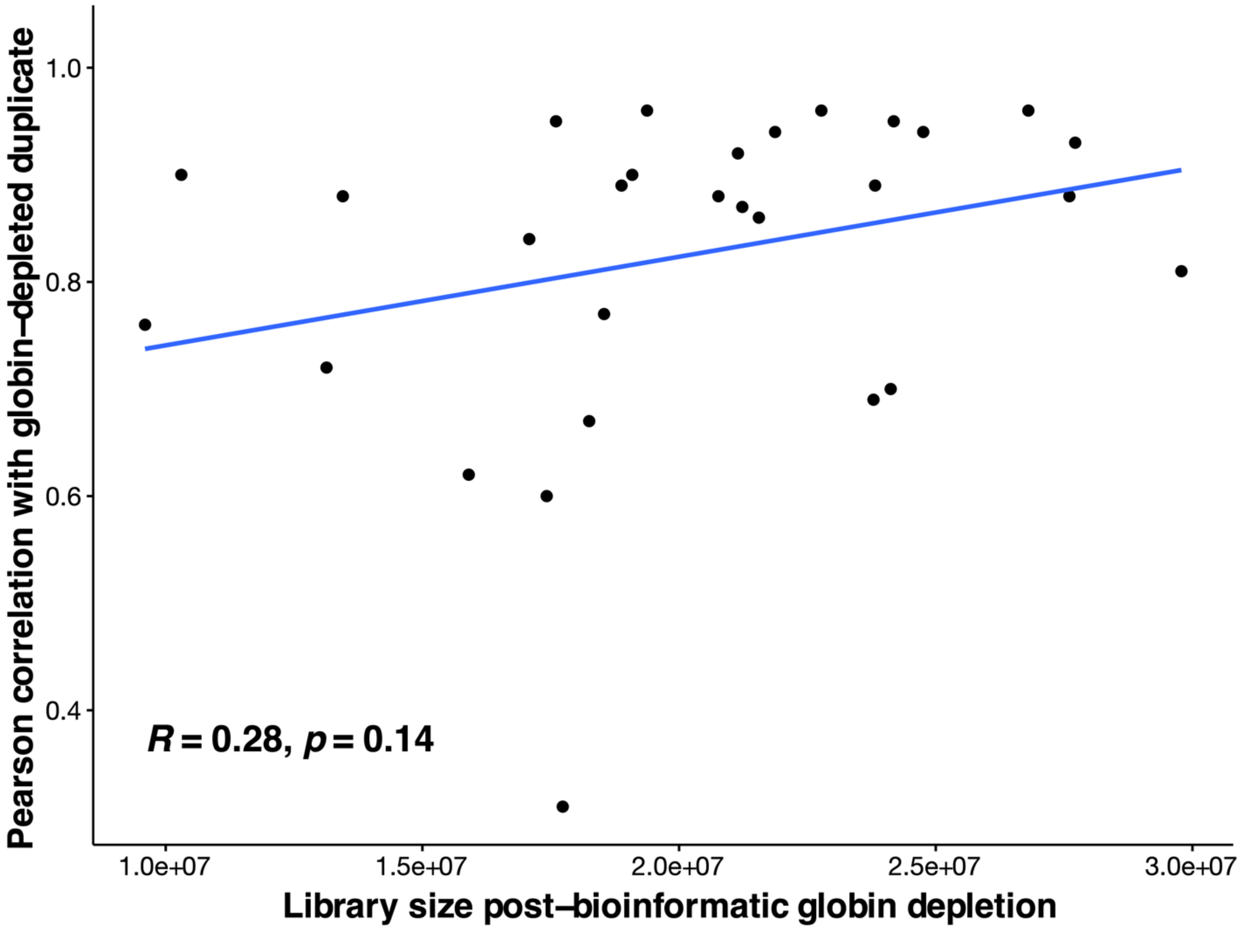

Supplementary Figure 3. Poor correlation between matched samples cannot be explained entirely by differences in library size. Pearson correlation between the library size of each sample following bioinformatic globin depletion and the Pearson correlation values derived from a correlation analysis of gene set variation analysis (GSVA) scores generated from 70 tuberculosis signatures between matched pairs of globin-depleted (n = 22) and non-depleted samples (n = 22). The blue line represents the fitted linear regression.
