# Supplementary material for: Identification and control for the effects of bioinformatic globin depletion on human RNA-seq differential expression analysis
Source: Sci Rep. 2023 Feb 1;13:1859. doi: 10.1038/s41598-023-28218-7 (PMC9892020; doi:10.1038/s41598-023-28218-7)
Supplement: Supplementary file 2 — Supplementary Information 2. [file 41598_2023_28218_MOESM2_ESM.docx]

| **Supplementary Table 1. Summary of samples and groups included in the RNA-sequencing analysis.** | | | | | |
| --- | --- | --- | --- | --- | --- |
| **Sex: n**  **Participants**  **Sample #** | **Median blood Haemoglobin (g/dL) [range]** | **Sample n by globin kit-depletion status** | **Median proportion reads mapping to haemoglobin genes [range]** | **Visit: sample n^1^** | **HIV-1 status** |
| Female: 8  32 samples | 12 [11.2–13.7]**^2^** | Non-depleted:  16 | 56% [41–67.2] | TB Diagnosis (D0): 8 | Positive |
|  |  |  |  | TB Post-treatment (M6): 8 |  |
|  |  | Globin kit-depleted:  16 | 0.27% [0.2–0.7] | TB Diagnosis (D0): 8 |  |
|  |  |  |  | TB Post-treatment (M6): 8 |  |
| Male: 8  26 samples | 14.45 [12.4–17.3]**^3^** | Non-depleted:  13 | 57.9% [35.8–80.3] | TB Diagnosis (D0): 3 | Positive |
|  |  |  |  | TB Post-treatment (M6): 3 |  |
|  |  |  |  | Asymptomatic contact: 3 | Negative |
|  |  |  |  | TB Diagnosis: 2 |  |
|  |  |  |  | TB Post-treatment: 2 |  |
|  |  | Globin kit-depleted:  13 | 0.33% [0.15–1.6] | TB Diagnosis (D0): 3 | Positive |
|  |  |  |  | Post-treatment (M6): 3 |  |
|  |  |  |  | Asymptomatic contact: 3 | Negative |
|  |  |  |  | TB Diagnosis: 2 |  |
|  |  |  |  | TB Post-treatment: 2 |  |
| **^1^**Coloured rows for visit column indicates samples used for paired analyses.  **^2^**D0, *n* = 5; M6, *n* = 7  **^3^**D0, *n* = 2; M6, *n* = 3 | | | | | |
